# Effect of protein binding substances on T1 times and the partition coefficient in contrast-enhanced cardiac magnetic resonance imaging

**DOI:** 10.1186/1532-429X-15-S1-E15

**Published:** 2013-01-30

**Authors:** Nadine Kawel, Francesco Santini, Tanja Haas, Johannes M Froehlich, Jens Bremerich

**Affiliations:** 1 Department of Radiology and Nuclear Medicine, University Hospital Basel, Basel, Switzerland; 2 Department of Radiological Physics, University Hospital Basel, Basel, Switzerland; 3 Scientific Affairs, Guerbet Switzerland, Zurich, Switzerland

## Background

Diffuse myocardial fibrosis is present in several cardiomyopathies. Contrast enhanced cardiac MR with T1 mapping enables quantification of diffuse fibrosis. T1 mapping is technically demanding since technical, physiological, and biochemical factors can interfere with T1 measurements. Contrast material dose, relaxivity, biodistribution, clearance, interaction with plasma proteins, and interference with co-medication must be taken into consideration. Gadobenate dimeglumine (Gd-BOPTA) has some weak protein binding capacities and higher molar relaxivity in plasma/blood resulting in shorter T1 times as compared to other extracellular gadolinium based contrast agents. To date it has not been assessed whether co-medication with other protein binding drugs alter myocardial T1 time. Therefore the aim of this study was to evaluate the interference of a typical protein binding drug (Ibuprofen) with Gd-BOPTA with respect to T1 times *in vitro* and *in vivo.*

## Methods

50 vials were prepared with different concentrations of gadobenate dimeglumine (0-16 mmol/l), Ibuprofen (0-4.85 mmol/l) and human serum albumin (0-4 g/l) in physiologic NaCl solution and imaged at 1.5T with a spin echo sequence at multiple TRs to measure T1 values and calculate relaxivities. 10 volunteers (5 men; 31±6.3 years) were imaged at 1.5T. T1 values for myocardium and blood pool were determined for various time points after administration of 0.15 mmol/kg gadobenate dimeglumine using a modified look-locker inversion-recovery sequence before and after administration of Ibuprofen over 24 hours. The partition coefficient was calculated as ΔR1myocardium/ΔR1blood, where R1=1/T1.

## Results

In vitro no significant correlation was found between relaxivity and Ibuprofen concentration, neither in absence (r=-0.15, p=0.40) nor in presence of albumine (r=-0.32, p=0.30). *In vivo* there was no significant difference in post contrast T1 times of myocardium and blood, respectively and also in the partition coefficient between exam 1 and 2 (p>0.05). There was an excellent correlation of T1 times of myocardium and blood, respectively between exam 1 and 2 (r=1.0, p<0.0001).

## Conclusions

Contrast enhanced T1 mapping with Gd-BOPTA is unaffected by co-medication with protein binding substances. Due to its excellent reproducibility it might be useful for follow-up exams.

## Funding

This study was sponsored in part by Guerbet Pharmaceutical Company Switzerland.

